# Phylogeny, Divergent Evolution, and Speciation of Sulfur-Oxidizing *Acidithiobacillus* Populations

**DOI:** 10.1186/s12864-019-5827-6

**Published:** 2019-05-30

**Authors:** Xian Zhang, Xueduan Liu, Liangzhi Li, Guanyun Wei, Danli Zhang, Yili Liang, Bo Miao

**Affiliations:** 1 Department of Occupational and Environmental Health, Xiangya School of Public Health, Central South University, Changsha, China; 2 School of Minerals Processing and Bioengineering, Central South University, Changsha, China; 3 Key Laboratory of Biometallurgy of Ministry of Education, Central South University, Changsha, China; 4 School of Life Sciences, Nantong University, Nantong, China; 5 Department of Biology, Taiyuan Normal University, Taiyuan, China

**Keywords:** *Acidithiobacillus*, Comparative genomics, Phylogeny, Divergent evolution

## Abstract

**Background:**

Habitats colonized by acidophiles as an ideal physical barrier may induce genetic exchange of microbial members within the common communities, but little is known about how species in extremely acidic environments diverge and evolve.

**Results:**

Using the acidophilic sulfur-oxidizer *Acidithiobacillus* as a case study, taxonomic reclassifications of many isolates provides novel insights into their phylogenetic lineage. Whole-genome-based comparisons were attempted to investigate the intra- and inter-species divergence. Recent studies clarified that functional and structural specificities of bacterial strains might provide opportunities for adaptive evolution responding to local environmental conditions. Acidophilic microorganisms play a key role in the acidification of natural waters and thus the formation of extremely acidic environments, and the feedbacks of the latter might confer the distinct evolutionary patterns of *Acidithiobacillus* spp. Varied horizontal gene transfer events occurred in different bacterial strains, probably resulting in the expansion of *Acidithiobacillus* genomes. Gene loss as another evolutionary force might cause the adaptive phenotypic diversity. A conceptual model for potential community-dependent evolutionary adaptation was thus proposed to illustrate the observed genome differentiation.

**Conclusions:**

Collectively, the findings shed light on the phylogeny and divergent evolution of *Acidithiobacillus* strains, and provided a useful reference for evolutionary studies of other extremophiles.

**Electronic supplementary material:**

The online version of this article (10.1186/s12864-019-5827-6) contains supplementary material, which is available to authorized users.

## Background

Knowledge about physiological and ecological constraints to organismal dispersal has extended our understanding of geographic barriers that limit gene flow across species in different regions. For macroorganisms, generally, dispersal barriers hinder the genetic exchange between species in different geographical areas, and local evolutionary processes such as genetic drift and econiche adaptation contribute to endemism [1], the equivalent of term ‘precinctive’ that refers to species in the restricted geographic location. To the contrary, microbial life including single-celled organisms are small-size and dispersed easily, and their biogeographical distributions are traditionally regarded to be a result of environmental selection (quoted by the old microbiological tenet ‘Everything is everywhere, but, the environment selects’) rather than dispersal limitation and stochasticity [2]. In recent years, however, considerable efforts have been attempted to elucidate a high degree of microbial endemicity [3–6]. Earlier studies have emphasized the importance of geographical isolation in the diversification and evolution of microorganisms on a global scale [7, 8]. Spatial separation, to a large extent, significantly affects the allopatric speciation in microbial world. In some isolated econiches, microbial species with low capacities to acquire alien genes, such as *Buchnera aphidicola*, was reported to exhibit an extreme genome stability [9], suggesting the effect of dispersal limitation on microbial speciation and evolution. Numerous studies pertaining to microbial endemicity have been made, yet knowledges about how geography influences divergence, evolution, and speciation of microorganisms remain elusive.

*Acidithiobacillus*, type genus of the family *Acidithiobacillaceae*, are recognized as sulfur-oxidizing acidophiles, and ubiquitously found in extremely acidic environments with heavy metal-laden and nutrient-deficient conditions, although these adverse settings are inhospitable to most life forms [10]. According to the public database List of Prokaryotic names with Standing in Nomenclature (LPSN, which is available at http://www.bacterio.net), several validated species of genus *Acidithiobacillus* have been described, including *Acidithiobacillus thiooxidans* (type species of this genus; formerly *Thiobacillus thiooxidans*) [11, 12], *Acidithiobacillus albertensis* (formerly *Thiobacillus albertis*) [13], *Acidithiobacillus caldus* (formerly *Thiobacillus caldus*) [14], *Acidithiobacillus ferrooxidans* (formerly *Thiobacillus ferrooxidans*) [15], *Acidithiobacillus ferrivorans* [16], *Acidithiobacillus ferridurans* [17], and *Acidithiobacillus ferriphilus* [18]. So far, numerous 16S rRNA gene sequences and draft/complete genome sequences of *Acidithiobacillus* spp. are available in public databases, thanks to the development of high-throughput sequencing technology.

Members of genus *Acidithiobacillus* are dominant in diverse sulfur-rich environments worldwide, and they are believed to play key roles in the biogeochemical cycle of sulfur and/or iron [19]. The metabolic activities of acidophiles in both pristine and anthropogenic sulfur-laden econiches contribute to the acidification of natural waters and the formation of extremely acidic habitats [20, 21]. Geochemical conditions of hyperacidic environments (pH < 3) are significantly different from that of surrounding regions, it might provide a potential geographic barrier that limits gene dispersal between acidophilic microorganisms, i.e., *Acidithiobacillus* populations, and other organisms in the surroundings. In other words, extremely acidic environments as a physical barrier might limit the access of indigenous genes to the global microbial gene pool, and hence increase the frequency of gene drift across the common biological communities. Here, we therefore hypothesized that spatial separation and geochemical isolation would cause the formation of separate ecosystems, which were stochastically colonized by various ancestral species, and a potential community-dependent evolutionary model was developed to delineate the allopatric divergence of *Acidithiobacillus* spp., which probably reflect the species-specific adaption to local environmental conditions.

## Methods

### Species selection used in this study

In the last several decades, a vast number of *Acidithiobacillus* strains have been isolated from a diverse range of sulfur-abundant environments worldwide [22], and plentiful 16S rRNA gene sequence dataset has been acquired on the basis of environmental sampling [23]. However, the taxonomic assignments of many of these strains and sequence clones are as yet unclear, which confuses the understanding of evolutionary lineage of *Acidithiobacillus* spp. Accordingly, it is necessary for these unclassified and cryptic species to conduct a more exhaustive revision of the phylogenetic taxon [21].

The genus (*Acidi*)*thiobacillus* is presently composed of seven identified species, dating back to 1922. In this study, the available genomes of *Acidithiobacillus* strains were downloaded from GenBank database, and 16S rRNA gene sequences of type strains were extracted from individual genomes using the RNAmmer 1.2 Server [24]. As listed in Additional file 1: Table S1, type strains contain *A. ferrooxidans* ATCC 23270^T^ (NC_011761), *A. caldus* ATCC 51756^T^ (NZ_CP005986), and *A. albertensis* DSM 14366^T^ (MOAD00000000). In addition, *A. thiooxidans* ATCC 19377^T^ (AFOH00000000) was included in this study. Given the lack of genomic sequence of *A. ferrivorans* DSM 22755^T^, strain SS3 (NC_015942) was used as a reference. In the absence of genomic sequences of *A. ferridurans* ATCC 33020^T^ and *A. ferriphilus* DSM 100412^T^, the partial 16S rRNA gene sequences were acquired according to LPSN, accession numbers of which were AJ278719 and KR905751, respectively. Apart from type strains, other existing complete/draft genomes of *Acidithiobacillus* strains deposited in public database were also used for the extraction of 16S rRNA gene sequences and then for the phylogenetic analysis. Referred to the length of above-mentioned sequences (ranging from 1,323 bp to 1,527 bp), 572 additional 16S rRNA gene sequences of *Acidithiobacillus* spp. were obtained from GenBank database, fitting the sequence length (ranging from 1,300 bp to 1,550 bp) and non-redundancy requirements.

### Analyses of phylogeny and taxonomy

Using a broader dataset that comprises up to 602 16S rRNA gene sequences of *Acidithiobacillus* spp. (Additional file 2: Table S2), sequence alignment was conducted using the online service MAFFT v7.402 [25] with a FFT-NS-2 method. The software Mesquite v3.51 was applied for the conversion of FASTA format to PHYLIP format. Given that saturation in substitutions could lead to incorrect phylogenetic inferences [26], saturation test was thus performed to evaluate the number of transitions and transversions against the Tamura-Nei (TN93) [27] genetic distance using DAMBE v5.2.73 [28], referred to a previous study [29]. Maximum-likelihood phylogeny was constructed using the online PhyML 3.0 [30] with the following settings: nucleotide substitution model, TN93; number of substitution rate categories, 6; and tree search algorithm, subtree pruning and regrafting. Visualization for 16S rRNA gene-based phylogenetic tree was performed using the program FigTree v1.4.3 (available at http://tree.bio.ed.ac.uk/software/figtree/).

As of July 2018, many genomes of *Acidithiobacillus* spp. have been released and deposited at the GenBank database, including genomes from five defined species and some other unclassified strains. *Acidithiobacillus* genomes have been automatically processed by NCBI prokaryotic genome annotation pipeline, such as gene prediction and functional annotation. In this study, 28 of bacterial genomes were included. Detailed genome information was illustrated in Additional file 1: Figure S1, such as accession numbers and genome sizes. Alignment-free phylogeny based on concatenated protein sequences of *Acidithiobacillus* genomes was constructed using the web server CVTree3 [31] with K-tuple length 6. Herein, *Achromobacter xylosoxidans* A8 was used as outgroup. The phylogenomic tree was then visualized using MEGA5.

Recently, average nucleotide identity (ANI) approach [32] was developed to substitute the conventional laboratory-based DNA-DNA hybridization, a gold standard for prokaryotic species identification. In our study, all 28 complete and/or draft genomes of *Acidithiobacillus* strains (Additional file 1: Figure S1) were used for the evaluation of their genomic similarity. Pairwise comparisons of bacterial ANI based on BLAST (ANIb) and MUMmer algorithm (ANIm) were performed using the online service JSpeciesWS [33] with default settings.

### Identification of orthologous and non-orthologous genes

To identify the putative orthologous and non-orthologous genes among *Acidithiobacillus* strains, BLASTP all-versus-all comparisons of entire amino acid sequences between pairs of *Acidithiobacillus* strains were conducted. In order to further explore the intra- and inter-species diversity of *Acidithiobacillus* spp., the computational tool Bacterial Pan Genome Analysis (BPGA) [34] was then applied to determine the orthologs and non-orthologs, including orthologous genes that were shared by all tested genomes or a subset of bacterial genomes, and strain-specific genes. In this procedure, we implemented the following parameters: *E*-value cut-off, 1e^-5^; and sequence identity cut-off, 50%. Giving the lineage-specific expansions of mobile genetic elements [35], these genes were excluded prior to the identification of orthologs. We finally performed the manual inspection and correction of all results. CVTree3 were employed to construct the phylogenetic tree, which was based on orthologous proteins shared by all *Acidithiobacillus* strains.

Functional assignments of core, accessory, and unique genes among *Acidithiobacillus* strains were performed via BLASTP alignment against specialized database, i.e., the extended Clusters of Orthologous Groups (COG) [36], with *E*-value cut-off set to 1e^-5^. The BLAST results were then manually checked based on the highest hit coverage value. Additionally, KEGG Automatic Annotation Server (KAAS) [37] was implemented to execute the automatically functional annotation of query amino acid sequences, with an assignment method of single-directional best hit. Statistical calculation was performed to identify the relative abundance of genes assigned to COG categories and KEGG pathways.

### Model extrapolation for *Acidithiobacillus* pan-genome

BPGA pipeline [34] was applied to extrapolate the pan-genome and core genome size based on 28 *Acidithiobacillus* genomes, as described in previous study [38]. To avoid the bias after any new genomes were added, random permutations were performed in the order of addition of genomes and medians were used to estimate the average number of pan-genomes and core genomes. In this procedure, the size of pan-genome and core genome were extrapolated by fitting the empirical power law equation [*Ps*(*n*) = *κn*^γ^] and exponential equation [*Fc*(*n*) = *κ*_*c*_exp(*-n*/*τ*_*c*_) + Ω] respectively, where *Ps*(*n*) and *Fc*(*n*) denote the calculated pan-genome and core genome sizes respectively, *n* is the number of sequenced genomes, and *k*, *γ*, *κ*_*c*_, *τ*_*c*_, and Ω are the fitting parameters. The exponent *γ* < 0 and 0 <*γ* < 1 indicate that *Acidithiobacillus* pan-genome is ‘closed’ and ‘open’, respectively.

### Calculation for gene turnover of *Acidithiobacillus* genomes

The program OrthoFinder v2.3.1 [39] with Markov cluster algorithm [40] and Diamond was applied to identify the orthogroups (herein referred as gene families) of *Acidithiobacillus* genomes. To detect the rates of gene gain and death, we performed the program BadiRate v1.35 [41] with a ‘GD-FR-CWP’ model by counting gain/loss events from the minimum number of family members in the phylogenetic nodes (inferred by the Wagner parsimony algorithm). Additionally, the topology of the whole-genome-based phylogeny was used as the reference tree. In the phylogenetic tree, each branch had its own turnover rate.

### Prediction of mobile genetic elements

Amino acid sequences of *Acidithiobacillus* strains were extracted from individual genomes using in-house Perl script, and putative transposable elements including transposon and insertion sequence (IS) were predicted using an updated version of online tool ISfinder [42] with an *E*-value cut-off of 1e^-5^. The web server tRNAscan-SE 2.0 [43] was used to search for the putative tRNA genes. Putative phage-associated genes in the sequenced *Acidithiobacillus* genomes were detected using the tool Prophinder [44] and BLASTP search against the ACLAME database of proteins in viruses and prophages [45] with the *E*-value threshold of 1e^-3^, as described previously [46]. Finally, all results above were manually checked.

## Results

### Phylogeny of Acidithiobacillus spp.

In our study, a set of 16S rRNA gene sequences were retrieved from GenBank database (Additional file 2: Table S2), according to certain criteria (see section Species selection used in this study). Saturation test was performed prior to the analysis of 16S rRNA gene-based phylogeny of *Acidithiobacillus* spp. As depicted in Additional file 1: Figure S2, transitions outnumbered transversions, suggesting that substitutions were not saturated and this dataset could be suitable for the subsequent construction of phylogenetic tree.

**Fig. 1:**
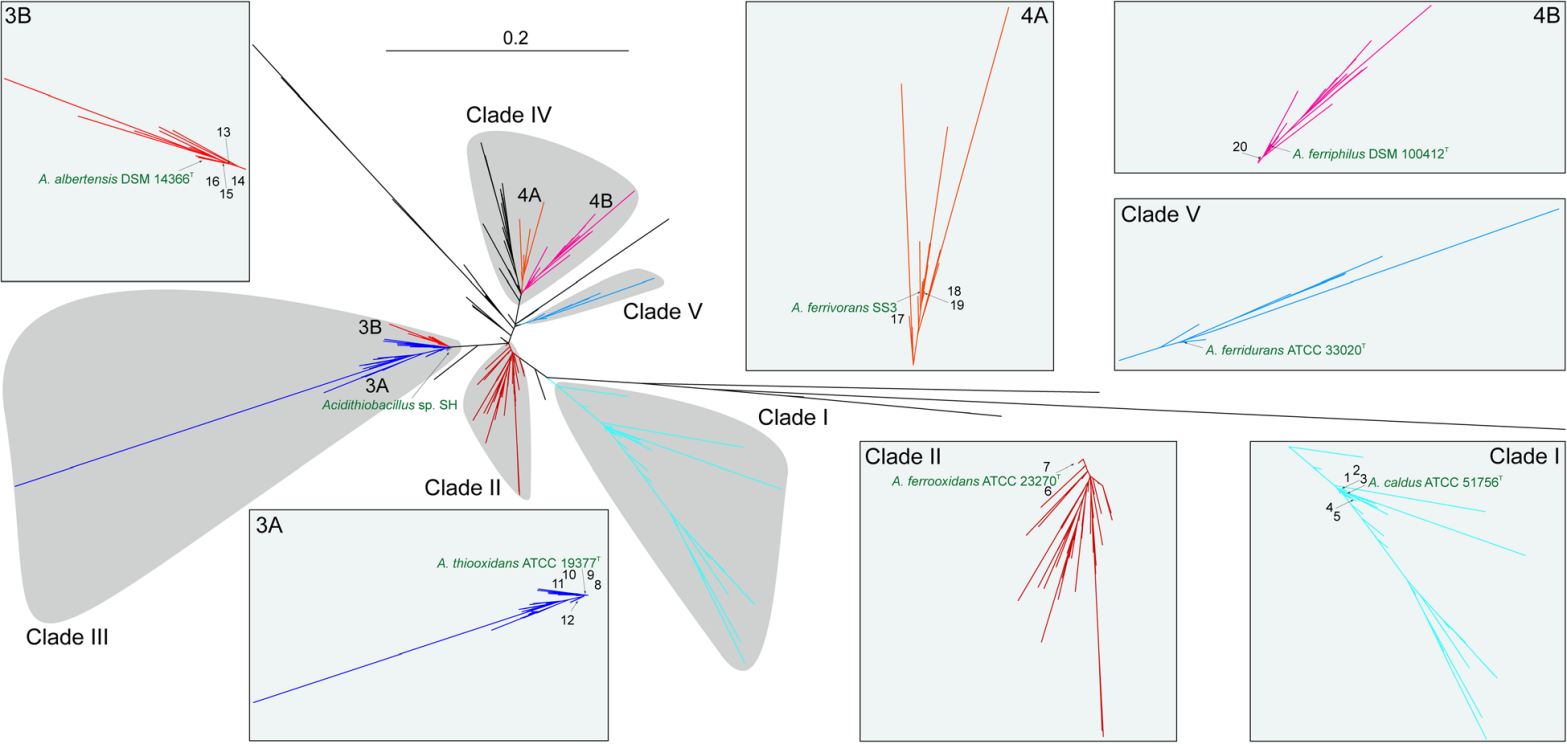
Maximum-likelihood (ML) phylogeny based on 16S rRNA gene sequences of *Acidithiobacillus* spp. Seven validated species are assigned into five clades, including *A. caldus* (Clade I), *A. ferrooxidans* (Clade II), *A. thiooxidans* (3A in Clade III), *A. albertensis* (3B in Clade III), *A. ferrivorans* (4A in Clade IV), *A. ferriphilus* (4B in Clade IV), and *A. ferridurans* (Clade V). These species are highlighted in different colors. Several strains of interest in various minor branches are also marked in this phylogenetic tree, including *A. caldus* strains DX (1), ZJ (2), ZBY (3), SM-1 (4), MTH-04 (5), *A. ferrooxidans* ATCC 53993 (6), *Acidithiobacillus* sp. GGI-221 (7), *A. thiooxidans* strains A01 (8), A02 (9), DMC (10), JYC-17 (11), BY-02 (12), ZBY (13), DXS-W (14), CLST (15), GD1-3 (16), *A. ferrivorans* strains YL15 (17), PRJEB5721 (18), CF27 (19), *A. ferrooxidans* BY0502 (20), and *Acidithiobacillus* sp. SH. Other branches representing unclear species are shown in black color. More details about these 602 16S rRNA gene sequences used in ML tree construction and their clade assignments are listed in Additional file 2: Table S2.

To evaluate the potential evolutionary relationships of all *Acidithiobacillus* strains and sequence clones, their available 16S rRNA genes as the marker were used for phylogeny. The resulting maximum-likelihood tree presented a coherent picture of *Acidithiobacillus* lineage (Fig. 1). Obviously, these strains and sequence clones were separately clustered into various distinctive clades, such as *A. caldus* ATCC 51756^T^ (Clade I), *A. ferrooxidans* ATCC 23270^T^ (Clade II), *A. thiooxidans* ATCC 19377^T^ (3A in Clade III), *A. albertensis* DSM 14366^T^ (3B in Clade III), *A. ferrivorans* SS3 (4A in Clade IV), *A. ferriphilus* DSM 100412^T^ (4B in Clade IV), and *A. ferridurans* ATCC 33020^T^ (Clade V). The finding was slightly different from an earlier study [21] , in which sequences from *A. thiooxidans*, *A. albertensis*, and *A. ferridurans* were gathered into the common clade. Additionally, other sister-clades possibly representing novel species or candidate phylotype, such as subclade within the *A. ferrivorans-A. ferriphilus* Clade IV (black colors), were shown in the branches of phylogenetic tree, suggesting a considerably underappreciated diversity within the genus *Acidithiobacillus*. Fascinatingly more, our study provided a revision for the isolates with unclassified and/or conflicting taxonomic assignments (Additional file 2: Table S2) via re-evaluating their phylogenetic relationships. As a result, many *Acidithiobacillus* spp. (e.g., *Acidithiobacillus* sp. GGI-221 within *A. ferrooxidans* Clade II) were assigned to the clades with recognized species, and the potential evolutionary relationships and classifications of several strains and sequence clones with known specific assignment (e.g., *A. ferrooxidans* BY0502 within *A. ferriphilus* subclade 4B) were reconfirmed according to our current 16S rRNA gene-based tree. Oddly, *A. thiooxidans* strains ZBY, DXS-W, CLST, and GD1-3 were apparently clustered into *A. albertensis* subclade 3B. Similarly, an earlier study revealed a high nucleotide sequence identity of up to 99.9% between *A. thiooxidans-A. albertensis* pairs [21], which exceeded both typical (97%) and conservative (98.7%) threshold values for species delineation [47, 48].

**Fig. 2:**
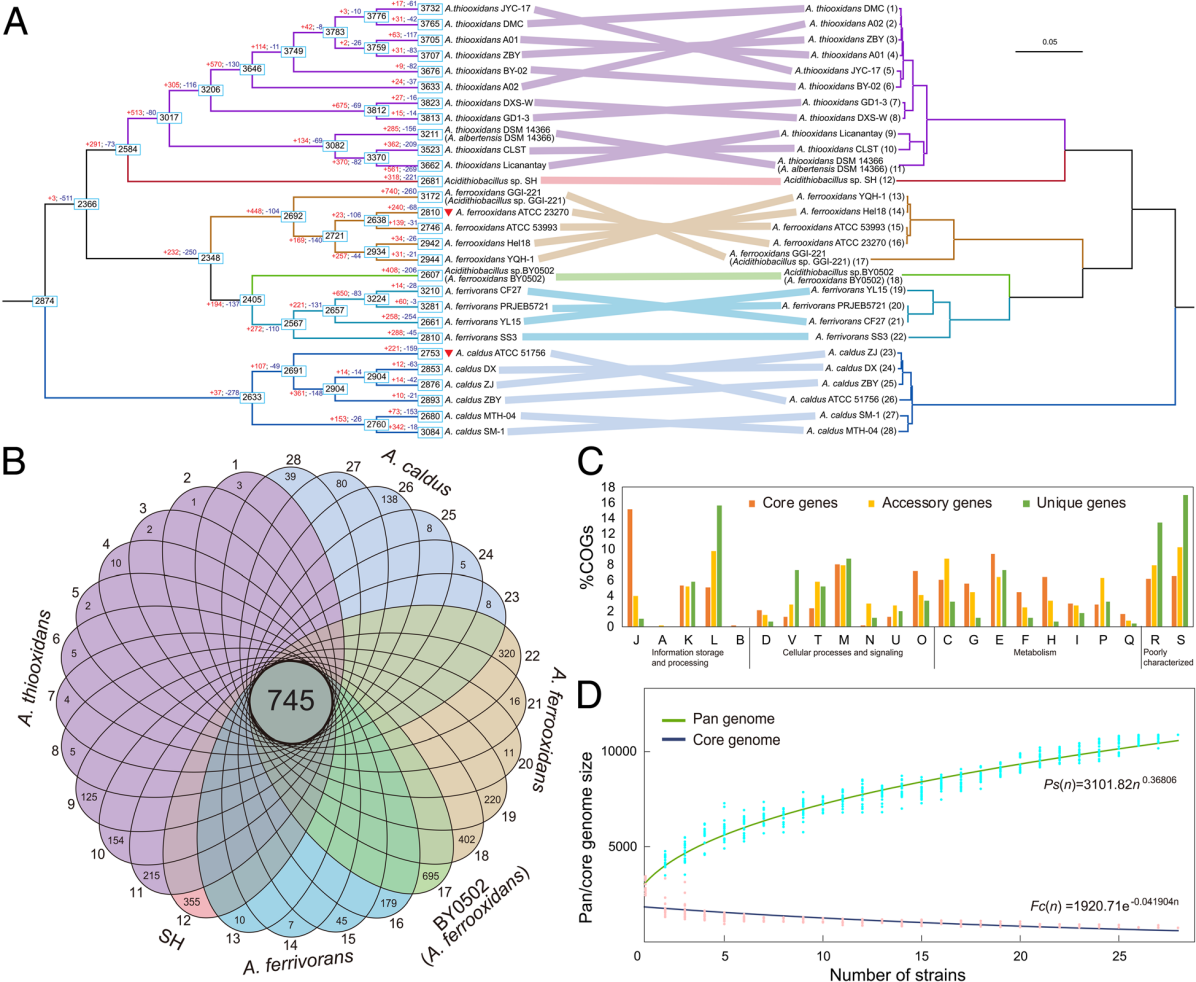
Comparative genomic analyses of 28 selected *Acidithiobacillus* strains. **a** Phylogeny based on concatenated proteins of *Acidithiobacillus* genomes and orthologous proteins shared by *Acidithiobacillus* spp. using CVTree3 with a composition vector approach. The topology of phylogenomic tree was shown, and *Achromobacter xylosoxidans* A8 was used as outgroup. Type strains are shown in red triangles. Specially, the redefined species are shown in the phylogenomic tree, and their former names can be also found in brackets. Numbers at internal nodes represent the number of possible ancestral genes as retrieved under the GD-FR-CWP model. Numbers on the branches and at the end of branches indicate the number of gain (red) and loss (blue) genes, and the extant counts of genes, respectively. For core genome-based phylogeny, the species are numbered in sequence. **b** Venn diagram representing the orthologous and non-orthologous genes of *Acidithiobacillus* spp. The number of orthologous genes shared by 28 *Acidithiobacillus* genomes and the strain-specific genes are shown in the center circle and petals, respectively. **c** COG assignments of core, accessory, and unique genes. Abbreviations: J, translation, ribosomal structure, and biogenesis; A, RNA processing and modification; K, transcription; L, replication, recombination, and repair; B, chromatin structure and dynamics; D, cell cycle control, cell division, chromosome partitioning; V, defense mechanisms; T, signal transduction mechanisms; M, cell wall/membrane/envelope biogenesis; N, cell motility; U, intracellular trafficking, secretion, and vesicular transport; O, posttranslational modification, protein turnover, chaperones; C, energy production and conversion; G, carbohydrate transport and metabolism; E, amino acid transport and metabolism; F, nucleotide transport and metabolism; H, coenzyme transport and metabolism; I, lipid transport and metabolism; P, inorganic ion transport and metabolism; Q, secondary metabolites biosynthesis, transport and catabolism; R, general function prediction only; S, function unknown. **d** Mathematical extrapolation for the estimation of size of *Acidithiobacillus* pan-genome and core genome. Detailed description for calculation is shown in section Model Extrapolation for *Acidithiobacillus* Pan-Genome.

A comprehensive strategy based on *Acidthiobacillus* genomes was used to improve the phylogenetic resolution and consolidate their evolutionary relationships. Based on 28 *Acidthiobacillus* genomes, these sequences were expected to be assigned into diverse clades (Fig. 2a). In this phylogenomic tree, strains of each species were clustered but apparently separated from that of the others. Similar to the 16S rRNA gene-based phylogeny, strain GGI-221 was likely to be phylogenetically affiliated to *A. ferrooxidans* species, and strain BY0502 might be a member of novel species rather than that of the current delineated species, i.e., *A. ferrooxidans*. Additionally, ANIb and ANIm values further supported the taxonomic reclassifications (Additional file 1: Figure S3). Notably, a striking finding was the affiliation of strain DSM 14366 (formerly belonged to *A. albertensis*). The values (≥ 96.62%) of ANIb and ANIm between DSM 14366 and each *A. thiooxidans* strains, accompanied by phylogenomic indicator, strongly indicated that strain DSM 14366 was likely to be affiliated to the type species *A. thiooxidans* instead of *A. albertensis*.

### Identification and functional assignments of orthologs and non-orthologs

Using a total of 28 available genomes, the pipeline BPGA was implemented to systematically explore the hereditary differentiation of *Acidthiobacillus* isolates. As demonstrated in Fig. 2b, inter- and intra-species diversity could be strongly reflected by the number of orthologs and non-orthologs. Intriguingly, many genes (745) were common in all *Acidthiobacillus* strains, probably indicating that these acidophiles were likely to share a suit of specialized genes contributing to the basic lifestyle and major phenotypic traits. Furthermore, the strain-specific genes were identified in each *Acidthiobacillus* genomes, showing the individual difference at the genome level. The rest genes shared by a subset of *Acidthiobacillus* strains was accessory genome, which was traditionally recognized to be responsible for species diversity and probably confer selective advantages such as econiche adaptation [49, 50].

Functional classifications of core, accessory, and unique genes among these *Acidthiobacillus* genomes were performed using BLAST algorithm against the COG database. Of these sequences, generally, most have no match in the current database, which was similar to many previous studies [51–54]. Apart from these sequences with no hits, the large proportion of genes (39.33%) in the core genome were predicted to pertain to the metabolic profiles (Fig. 2c), highlighting the potential contribution of metabolism-related genes in mediating the autotrophic lifestyle of *Acidthiobacillus* spp. KAAS annotation (Additional file 1: Table S3) revealed that genes involved in carbohydrate metabolism (81), amino acid metabolism (80), metabolism of cofactors and vitamins (57), and energy metabolism (55) outnumbered other metabolism-associated genes. Further inspection revealed that the three most abundant genes were assigned to COG categories [J] (translation, ribosomal structure, and biogenesis), [E] (amino acid transport and metabolism), and [M] (cell wall/membrane/envelope biogenesis). As for accessory and unique genes, however, most of them were related to COG categories [L] (replication, recombination, and repair); The finding was frequently found in other similar studies [52, 55].

### Mathematical extrapolation for estimating the size of *Acidithiobacillus* pan-genome

Comparative analysis showed that the relatively small proportion of protein-coding genes (18.6% - 27.5%) were assigned to core genome. Additionally, the inconsistency between these two phylogenies based on whole-genome and core genome was identified (Fig. 2a), which provided an indication that abundant exogenous genes introduced by genetic exchange might contribute to genome plasticity. The inference, to a large extent, could be explained by a well counter-example about *B. aphidicola*, in which the most extreme genome stability was observed because its genome has undergone few events of chromosome rearrangements, or gene acquisitions in the past 50 to 70 million years [56].

Due to its genome expansion, in theory, novel genes would be introduced into *Acidithiobacillus* genomes with the new sequenced genome. To evaluate the size of *Acidithiobacillus* pan-genome, mathematical extrapolation based on the present 28 genomes was performed using the pipeline BPGA (Fig. 2d). Exponential equation [*Fc*(*n*) = 1920.71*e*^-0.041904n^] indicated that the extrapolated curve following a gentle slope reached a minimum of 745 after the 28th *Acidithiobacillus* genome was added into the dataset. Furthermore, empirical power law equation [*Ps*(*n*) = 3101.82n^0.36806^] revealed the *Acidithiobacillus* pan-genome with an average parameter of 0.36806. The exponent 1 > γ > 0, according to the Heaps’ law [57], hinted that its size was increasing and unbounded. Taken together, it was inferred that frequent lateral exchange of genetic material might dramatically expand the gene content of bacterial genomes, and promote the environmental adaptability of *Acidithiobacillus* isolates.

### Potential forces driving genome evolution of *Acidithiobacillus* strains

Adaptive evolution of microbial genomes is generally regarded to be driven by several genetic events, such as duplication, rearrangement, acquisition, and loss of genes and genome segments [58, 59]. Especially, many studies have discussed the potential roles of gene gain and loss in genome evolution in response to local environmental conditions [46, 52, 55, 60]. Both gene gain and loss are thought to be two key adaptive mechanisms that have great potential to drive genome evolution of microbial species, but their relative importance in shaping the content of microbial genomes and driving microbial evolution and speciation has been unclear yet.

To investigate the potential evolutionary forces, gene turnover rates of *Acidithiobacillus* genomes were first evaluated, followed by the prediction of mobile genetic elements. A subsequent analysis that relates the comparison of genomic regions of interest (such as genes associated with metabolic profiles) to the presence or absence of mobile genetic elements in the genomic neighborhoods may facilitate the deduction of evolutionary mechanisms that drive adaptive evolution and diversification of microbial genomes.

### Gene turnover potentially contributing to genome differentiation

Gene families in *Acidithiobacillus* genomes were classified as orthogroups and listed in Additional file 1: Table S4. To obtain an overview of the evolutionary dynamics of gene families, the ancestral gene set profiles were constructed at all nodes of the phylogenomic tree among these 28 fully sequenced strains (Fig. 2a). Variation occurred among *Acidithiobacillus* genomes in terms of the total gene numbers. On a global scale, numerous gain/loss events were frequently identified. Starting from a common ancestor, divergent evolution occurred between *A. caldus* strains and the others in an earlier time, with a dramatic gene decrease. Nevertheless, there was a global gene increase in many of branches. In short, many divergences among *Acidithiobacillus* strains were observed in the whole evolutionary process, accompanied by diverse events of both gene gain and loss. The data presented here hinted that frequent gene turnover might confer selective advantages to acidophiles in acid environments, probably contributing to the observed genome differentiation.

### Prediction of mobile genetic elements

Gene acquisition is usually accompanied by the events of horizontal gene transfer (HGT), which frequently occur in microorganisms and are beneficial for microbial species to rapidly adapt to changing environments [58]. A significant part of HGT is facilitated by mobile genetic elements, and is generally characterized by some signatures in microbial genomes, such as integration sites often related to tRNA genes, abnormal GC contents, or varied codon usage [61–63]. Accordingly, transposases and integrases were predicted and classified using the platform ISFinder (Additional file 3: Table S5). Results showed that putative transposable elements Tn3, ISL3, IS91, and IS1595 were present and abundant in most of *Acidithiobacillus* genomes. While many genes were assigned to various IS families, some transposable elements were identified unique in certain *Acidithiobacillus* strains, such as IS51 in *A. thiooxidans* Licanantay, and IS605 in several *A. thiooxidans* strains.

Putative phage-associated genes were identified in all *Acidithiobacillus* genomes using the server Prophinder, accompanied by BLASTP search against the proteins in viruses and prophages of ACLAME database. In our study, many predicted phage-associated genes were dispersed over certain genomic regions within *A*. *caldus* strains SM-1, MTH-04, and ATCC 51756, *A. ferrooxidans* ATCC 23270, and *A. thiooxidans* DSM 14366 (Additional file 4: Table S6), probably suggesting that these strains might been targeted by phage via infection. Referred to the previous studies [46, 64], especially, a gene cluster composed of several phage-associated genes in *A. caldus* ATCC 51756 (chromosome: 100435-143772) might be a fragment of the putative prophage, since some DNA packaging- (phage terminase large subunit and portal protein), head-, tail-, and cell lysis-associated genes were identified. However, genes related to small subunit of the phage terminase and phage DNA replication were absent in this genome. In addition, genes encoding putative prophage DNA integration, phage DNA replication, and some phage with unknown functions were predicted to be scattered throughout the genomic regions of *Acidithiobacillus* strains via comparison to the ACLAME database.

### Comparisons of genomic regions of interest in Acidithiobacillus spp.

Comparative genomics has facilitated the systematical investigation regarding the intra- and inter-species diversity. Comparisons of genomic regions of interest, accompanied by the prediction of putative mobile genetic elements, probably allow the identification of potential driving forces of genome evolution. Since metabolism-related genes accounted for a large proportion of bacterial genomes, we thus focused on the comparison of inferred metabolic profiles.

**Fig. 3:**
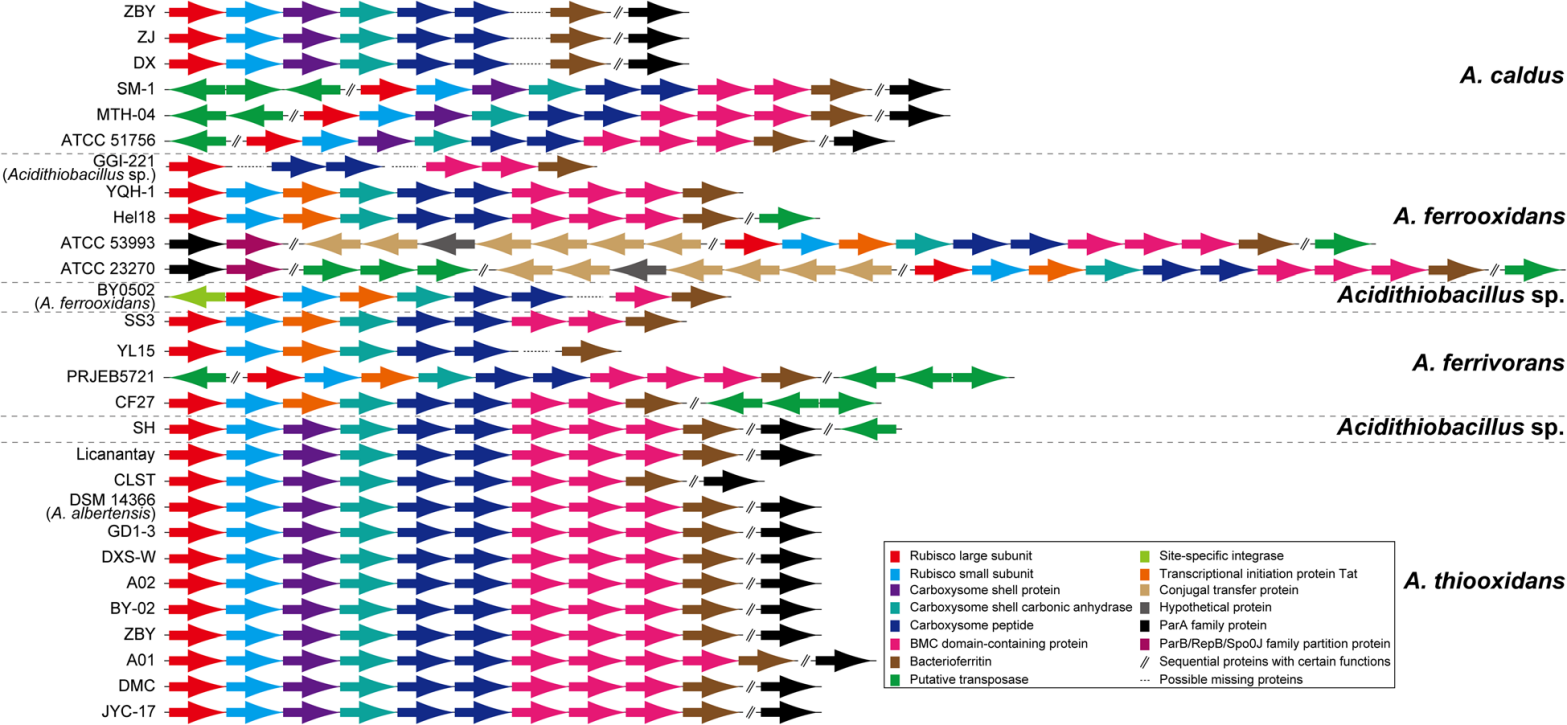
Genomic organization of the gene clusters related to carboxysomes in *Acidithiobacillus* strains. As depicted in this figure, the order and orientation of each gene are presented. Varied functional genes are shown in different colors. More details for gene information are shown in Additional file 4: Table S6.

Carboxysome, a cytoplasmic and polyhedral bacterial microcompartment (BMC), has been traditionally thought to be a central part of the carbon-concentrating mechanism, which was greatly useful for increasing the fixation of external carbon in cyanobacteria and some chemoautotrophs [65]. In our study, a gene cluster potentially associated with carboxysome was widespread in the obligate chemolithotroph *Acidithiobacillus*. Further inspection showed that the genomic organization of carboxysome gene clusters was similar in all *Acidithiobacillus* strains (Fig. 3 and Additional file 5: Table S7), mainly including large and small subunits of ribulose1,5-bisphosphate carboxylase/oxygenase (RubisCO), carboxysome shell protein (or transcriptional initiation protein Tat in *A. ferrooxidans* strains, *Acidithiobacillus* sp. BY0502, and *A. ferrivorans* strains), carboxysome shell carbonic anhydrase (CA; ε-class) [66], carboxysome peptides A and B, several BMC domain-containing proteins, and bacterioferritin. Despite their similar gene content, order, and orientation, fascinatingly, various putative transposases or site-specific integrases were predicted to be in the genomic neighborhoods (both upstream and downstream; Fig. 3). The similar observations have been documented in some other acidophiles inhabiting the acid environments, such as *Ferroplasma acidarmanus* [67] and *Ferrovum* sp. [46]. Other signatures of HGT including phage-associated genes and genes related to the type IV secretion system were further identified. No putative phage-associated genes were found in these genomic neighborhoods, but a set of genes encoding the components of putative type IV secretion system was predicted in *A. ferrooxidans* strains ATCC 23270 and ATCC 53993 (Additional file 5: Table S7). In addition, other characteristic function associated with plasmid partition system was predicted. Obviously, the ParAB partition system was in the upstream of carboxysome-associated gene clusters within *A. ferrooxidans* strains ATCC 23270 and ATCC 53993, while a truncated ParAB partition system, which lacked the ParB family partition protein, was found in the downstream of genomic region related to carboxysome within *A. caldus* strains, *Acidithiobacillus* sp. SH, and *A. thiooxidans* strains. As reported in the previous studies, the ParAB partition system was not only responsible for the segregation of low copy number plasmids [46], but also involved in the bacterial chromosome segregation [68]. So far, some *Acidithiobacillus* strains were documented to harbor at least one plasmid, including *A. ferrivorans* PRJEB5721, *A. caldus* strains ATCC 51756, SM-1, and MTH-04, although whether the plasmid existed in the others remains unclear.

Nitrogenase complex has been recognized to be the key enzyme directing the fixation of molecular nitrogen. In our study, a suit of *nif*-genes encoding the components of putative nitrogenase complex, such as MoFe cofactor biosynthesis proteins NifXNE and nitrogenase structural subunits NifKDH, was predicted to be present in *A. ferrooxidans* strains except for strain GGI-221, of which several genes were absent probably due to the potential frameshift (Additional file 5: Table S7). The identical genomic organization were also observed in all *A. ferrivorans* strains. However, no *nif*-genes were identified in the genomes of other *Acidithiobacillus* spp. Interestingly, a gene encoding putative transposase was inserted into the nitrogenase-associated gene cluster of *A. ferrivorans* strains CF27 and PRJEB5721. Additionally, an integrase-encoding gene was identified in the genomic neighborhood of *A. ferrooxidans* strains ATCC 23270 and ATCC 53993. In spite of these findings, the conserved genomic segments associated with nitrogenase complex strongly indicated that these *nif*-genes in all strains of *A. ferrooxidans* and *A. ferrivorans* were likely to be vertically inherited from the common ancestral genomes, and these strains with putative mobile genetic elements might undergo potential HGT events to recruit novel hereditable character instead of nitrogen-fixing ability. From another point of view, the absence of nitrogenase-associated gene cluster in other *Acidithiobacillus* isolates was likely to be the consequence of gene loss in the common ancestor of *Acidithiobacillus* spp. A similar observation for iron-oxidizing acidophile *L. ferriphilum* was reported in a previous study, in which homologous genes involved in nitrogenase complex were absent in strains DX and ML-04 [53].

## Discussion

The genus *Acidithiobacillus* has been currently recognized to be composed of seven validated species. Using a full set of 16S rRNA gene sequence data collected from the public database, the sampled species-level phylogeny was constructed to extrapolate the potential evolutionary lineage of *Acidithiobacillus* spp. Similar to a recent study [21], the current results revealed a significant diversity of genus *Acidithiobacillus*, suggesting that many underappreciated species within this genus remain to be recognized and redefined. In the past, provisional recognition of a number of *Acidithiobacillus* species, such as *A. concretivorus* [69–71] and *A. cuprithermicus* [72], has occurred, but some of them have often been questioned with regard to the validity of newly proposed ‘species’ [73]. Our current study provided additional evidence to revise the taxon of an *A. cuprithermicus* strain (Additional file 2: Table S2). In addition, many unclassified *Acidithiobacillus* spp., such as *Acidithiobacillus* sp. GGI-221, and strains belonging to identified species were re-evaluated (Fig. 1). Especially, the debatable *A. ferrooxidans* BY0502 and *A. albertensis* DSM 14366 were redefined, according to a polyphasic taxonomic study including 16S rRNA gene- and whole-genome-based phylogeny, and ANI approach. The findings presented here indicated that strain BY0502 might be phylogenetically affiliated to *A. ferriphilus* instead of the currently proposed *A. ferrooxidans*. However, more robust evidence based on genomic data should be provided to support the taxonomic assignment in the future study. As for strain DSM 14366, it has been previously proposed to be a member of species *A. albertensis*. Although physiologically similar to *A. thiooxidans*, this species was reported to be distinguished by harboring the glycocalyx- and flagella-associated genes [12]. And in fact, our previous studies have reported the presence of putative flagella-related genes in *A. thiooxidans* strains [10, 66]. Accordingly, the limited evidence might not support the controversially proposed *A. albertensis*. However, subspecies-level analyses remain to be attempted to improve the resolution of *Acidithiobacillus* phylogeny and further distinguish the ecologically distinct organisms with closely related taxa.

Comparative genomics has yielded insights into the genetic diversity of microbial genomes. In this study, functional assignments highlighted the relatively high proportion of accessory and strain-specific genes associated with COG category [L] (Fig. 2c), suggesting that these genes might confer adaptive advantage to harsh eco-environments, as high concentrations of toxic substances in acid environments, such as heavy metals, easily caused the DNA injury [74]. Additionally, a set of 745 genes common in all 28 *Acidithiobacillus* strains were identified (Fig. 2b). Although mathematical model showed that extrapolated curve has reached a trough (Fig 2d), this core set of genes might not be equal to the theoretically minimized genome of sulfur-oxidizing *Acidithiobacillus* populations. As stated by Koonin [75], the definition of ‘minimal gene-set’ should be associated with the local environmental conditions under which these genes were necessary and sufficient for survival and proliferation of any given organisms. Despite this, the presence of a set of core genes in bacterial genomes was testament to the conservative nature of long-term evolution [76]. Given these similar eco-environments where *Acidithiobacillus* strains inhabit, a backbone of conserved genes might be necessary for sustaining a functional cell. These essential genes might evolve from the last universal common ancestor in multiple ways, and endow bacterial species with opportunities to adapt to environmental perturbations. It might be reasonable that a large proportion of core genes were assigned to COG categories [J], [E], and [M], in light of the following possible explanations: (i) genes involved in translation were conserved in all cellular life forms [75]; (ii) efficient uptake of nutrients was essential for basic lifestyle of microorganisms [77]; and (iii) distinctive structural and functional characteristics were developed to cope with harsh environmental conditions such as extremely acidic settings [10], since specialized cellular structures and functions, e.g., highly impermeable membranes, selective outer membrane porin proteins, reversed membrane potential, and active proton pumping, were benefit for maintaining a stable pH gradient and thus for supporting the growth of acidophilic microorganisms at low pH [78]. In an earlier study, pH value was observed to selectively influence the expression of certain genes involved in key metabolisms in some iron/sulfur-oxidizing microorganisms [79]. Combined with comparative genomics in this study, it should be an interesting prospect to design other omics-oriented studies, such as transcriptomics and proteomics, in the future works to explore the potential pH homeostatic mechanisms of acidophiles.

Since the frequent gene turnover among *Acidithiobacillus* strains might contribute to their genetic diversity (Fig. 2a), further studies targeted the issues that what and how adaptive evolutionary forces influenced the gene repertoires of bacterial genomes. Focusing on genomic regions related to metabolic profiles, we found that several signatures of mobilization (transposases, integrases, phage-related genes, integration sites) and conjugation (the type IV secretion system) were scattered throughout the genomic regions associated with carboxysome, probably providing a coherent picture of the prevalence of varied HGT events. RubisCO, the key enzyme in carbon fixation, might allow the obligate lithoautotroph *Hydrogenovibrio marinus* MH-110 to adapt well to different CO_2_ concentrations [80]. And carboxysome-associated CA was reported to elevate the concentrations of carbon dioxide in the surrounding of Rubisco via effectively converting the accumulated cytosolic bicarbonate into CO_2_ [81]. Genes arose by HGT coupled with functional recruitments in order to cope with the harsh environmental conditions, indicating the high genome plasticity. However, the findings revealed that the absence of nitrogenase-associated genes in *A. thiooxidans* strains, *A. caldus* strains, *Acidithiobacillus* sp. strains BY0502 and SH might be caused by gene loss in the common ancestral genomes of *Acidithiobacillus* strains. Currently, the increasing genomic data deluge in public databases has been revealing an unexpected perspective of gene loss as an effective means of hereditary variation that potentially resulted in adaptive phenotypic diversity [82]. Referred to a classical theory, i.e., the Black Queen Hypothesis [83], a community-dependent evolutionary pattern was thus proposed to explain the observed loss of genomic segments. In the context of deficient nitrogen source in acid eco-environments [84], some microbial members in the common communities should make a ‘compromise’ to optimally utilize the limited resources of the entire communities. The fixation of externally-derived nitrogen might be partitioned into a small fraction of co-existing diazotrophic species, such as *A. ferrooxidans* and *A. ferrivorans*. The product of their metabolic activities may provide alternative nitrogen compounds, such as ammonium, for supporting the nitrogen supply of other co-occurring members without the nitrogen-fixing abilities.

**Fig. 4:**
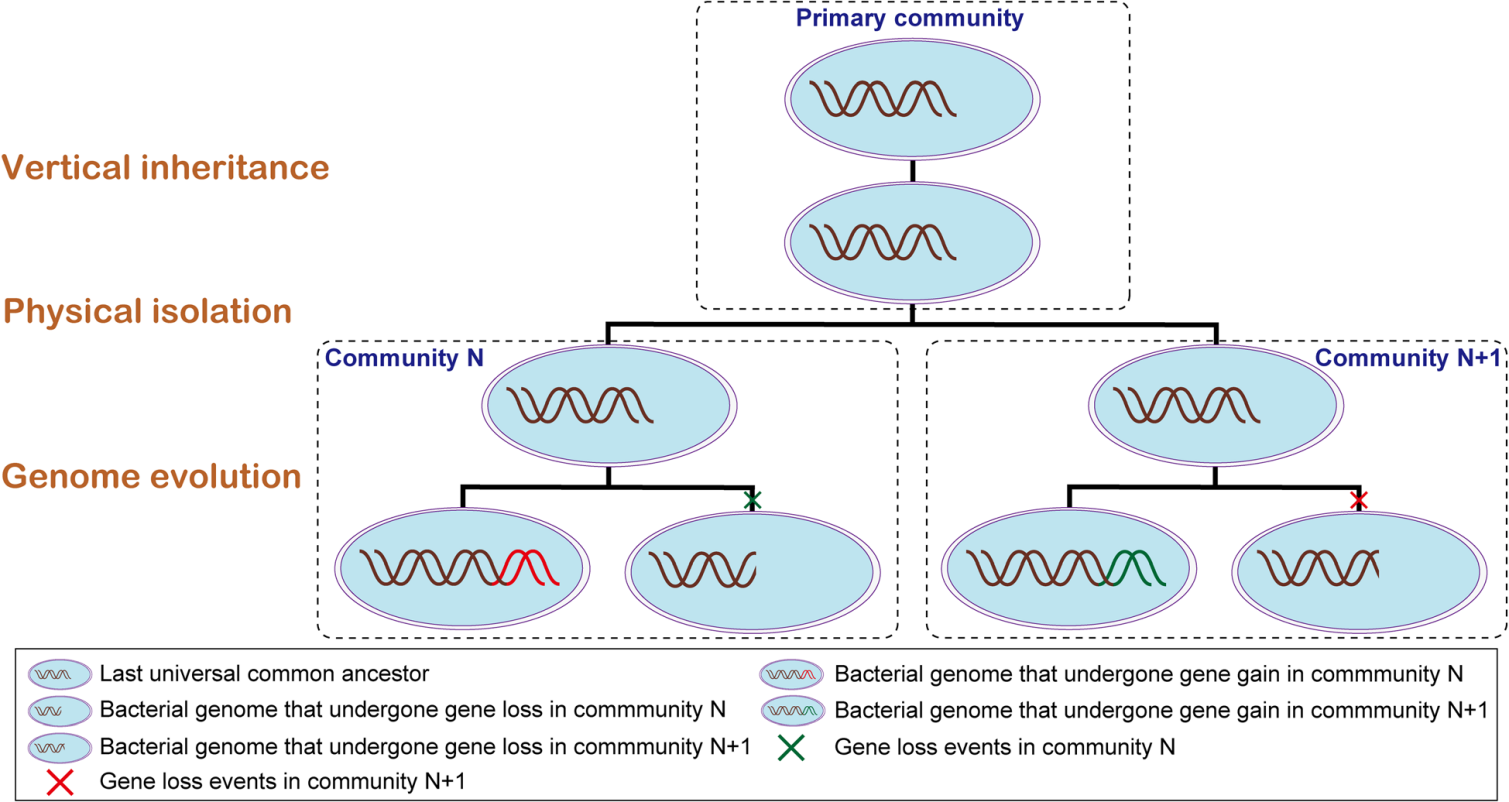
Schematic diagram depicting the potential community-dependent evolutionary model of *Acidithiobacillus* population. Extremely acidic environments as potential physical barrier might contribute to geographical isolation. Varied gene flow occurs in isolated microbial communities of ecological niches around the world. Gene gain events via various horizontal gene transfer expand the gene repertoires of bacterial genomes, and gain loss events dependent on microbial community increase cellular economization, thereby resulting in the allopatric speciation.

Taken together, a potential evolutionary model for *Acidithiobacillus* strains was thus proposed to extrapolate the hereditary differentiation patterns in response to the local environmental conditions (Fig. 4). Extremely acidic environments as a possible natural barrier might provide few opportunities for indigenous species to access the gene pool in the surrounding environments, but prompt the gene flow across these acidophilic microorganisms in the relatively isolated regions, thereby indicating that the evolution of given species might depend on other members in a common community. The essential genes in these acidophiles have vertically inherited from ancestral genomes to support their basic lifestyle, and meanwhile evolved the adaptive evolutionary mechanisms responding to the changing environments via various genetic events including gene gain and/or loss. Gene gain events mediated by transfer, transduction, and conjugation may extend the gene repertoires of microbial genomes and potentially recruit novel functionalities, and genome reduction dependent on community functions could economize the nutrient requirements under the resource-deficient conditions, resulting in the observed allopatric speciation.

## Conclusions

Phylogeny using both 16S rRNA gene and genome sequences expanded our understanding of the genetic diversity of *Acidithiobacillus* populations. Redefinitions of many questioned species provided new insights into the taxonomic assignments of bacterial species.

A recent study revealed the observation that presented a co-evolution pattern of acidophiles and their acidic habitats [20]. The metabolic activities of acidophilic microorganisms might contribute to the formation of extremely acidic environments, and the feedbacks of these existing harsh eco-environments, in turn, could enhance the exchange frequencies of genetic material within the members of entire microbial communities, thereby resulting in the habitat-driven genome evolution and speciation. Potential evolutionary forces were discussed in this study to further highlight the roles of gene gain and loss in genome evolution, and the donors and recipients of genes were suspected to be derived from the co-occurring members of microbial communities. Gene gain by HGT was doubtlessly recognized as an efficient way to expand the gene repertoire of given species, and gene loss in some ex-existing members might be a good choice to economize the limited resources of the whole community [83, 85]. A case study of genome reduction involved in nitrogen fixation may advance our current knowledge of community-dependent evolutionary adaptation of *Acidithiobacillus* isolates. Acidophilic microorganisms with a diazotrophic lifestyle act as pioneers to colonize new habitats, the later colonizers might increase cellular economization via gene loss to rationally utilize the limited resources, thereby resulting in adaptive evolution and genome differentiation within the whole community.

## Additional files

Additional file 1: **Figure S1.** Geographic distributions and genome attributes of *Acidithiobacillus* strains used for the construction of genome-based phylogeny and the calculation of average nucleotide identity. **Figure S2.** Substitution pattern of 16S rRNA genes used for phylogenetic tree. The number of transitions (cross) and transversions (triangle) against the TN93 distance is shown in different colors. Each point in individual sites indicates a pairwise comparison between two of taxa. **Figure S3.** Calculations of ANIb (upper half) and ANIm (lower half) for *Acidithiobacillus* strains. The values of ANI above the threshold for species delineation (95%) are highlighted. **Table S1.** Statistics for the number of RNA (rRNA and tRNA) in *Acidithiobacillus* (*A.*) strains with available genomes. **Table S3.** Functional classifications of the common genes shared by *Acidithiobacillus* strains using an online platform KAAS. **Table S4.** Summary for gene families in the genomes of *Acidithiobacillus* strains, including *A. thiooxidans* (formerly *A. albertensis*) DSM 14366 (1), *A. caldus* strains ATCC 51756 (2), DX (3), MTH-04 (4), SM-1 (5), ZBY (6), ZJ (7), GGI-221 within *A. ferrooxidans* (formerly *Acidithiobacillus* sp.) GGI-221 (8), *Acidithiobacillus* sp. SH (9), *A. ferrooxidans* strains ATCC 23270 (10), ATCC 53993 (11), Hel18 (12), YQH-1 (13), *Acidithiobacillus* sp. (formerly *A. ferrooxidans*) BY0502 (14), *A. ferrivorans* strains CF27 (15), PRJEB5721 (16), SS3 (17), YL15 (18), *A. thiooxidans* strains A01 (19), A02 (20), BY-02 (21), CLST (22), DMC (23), DXS-W (24), GD1-3 (25), JYC-17 (25), Licanantay (27), and ZBY (28). (DOC 37024 kb)

Additional file 2: **Table S2.** General properties of 16S rRNA genes of *Acidithiobacillus* isolates and clones available in the public database. (XLSX 33 kb)

Additional file 3: **Table S5.** Statistics for puative transposases in *Acidithiobacillus* genomes using the online tool ISFinder. (XLSX 13 kb)

Additional file 4: **Table S6.** Prediction of putative phage-associated genes in the genomes of *Acidithiobacillus* strains, including *A. caldus* strains SM-1, MTH-04, and ATCC 51756, *A. ferrooxidans* ATCC 23270, and *A. thiooxidans* DSM 14366. (XLSX 38 kb)

Additional file 5: **Table S7.** Gene content of genomic regions associated with carboxysome and nitrogenase in *Acidithiobacillus* strains. (XLSX 26 kb)

## Data Availability

The raw dataset, including 16S rRNA gene sequences and genomic sequences of *Acidithibacillus* spp., was available and download from the public database National Center for Biotechnology Information. All data supporting the findings of our study can be found within the manuscript and additional file tables.

## Abbreviations

ANI: Average nucleotide identity
ANIb: ANI based on BLAST algorithm
ANIm: ANI based on MUMmer algorithm
BMC: Microcompartment
BPGA: Bacterial Pan Genome Analysis
CA: Carbonic anhydrase
COG: Clusters of Orthologous Groups
HGT: Horizontal gene transfer
IS: Insertion sequence
KAAS: KEGG Automatic Annotation Server
LPSN: List of Prokaryotic names with Standing in Nomenclature
RubisCO: Ribulose1,5-bisphosphate carboxylase/oxygenase
TN93: Tamura-Nei
