# Supplementary material for: Phylogeny, Divergent Evolution, and Speciation of Sulfur-Oxidizing Acidithiobacillus Populations
Source: BMC Genomics. 2019 May 30;20:438. doi: 10.1186/s12864-019-5827-6 (PMC6543593; doi:10.1186/s12864-019-5827-6)
Supplement: Supplementary file 1 — Figure S1. Geographic distributions and genome attributes of Acidithiobacillus strains used for the construction of genome-based phylogeny and the calculation of average nucleotide identity. Figure S2. Substitution pattern of 16S rRNA genes used for phylogenetic tree. The number of transitions (cross) and transversions (triangle) against the TN93 distance is shown in different colors. Each point in individual sites indicates a pairwise comparison between two of taxa. Figure S3. Calculations of ANIb (upper half) and ANIm (lower half) for Acidithiobacillus strains. The values of ANI above the threshold for species delineation (95%) are highlighted. Table S1. Statistics for the number of RNA (rRNA and tRNA) in Acidithiobacillus (A.) strains with available genomes. Table S3. Functional classifications of the common genes shared by Acidithiobacillus strains using an online platform KAAS. Table S4. Summary for gene families in the genomes of Acidithiobacillus strains, including A. thiooxidans (formerly A. albertensis) DSM 14366 (1), A. caldus strains ATCC 51756 (2), DX (3), MTH-04 (4), SM-1 (5), ZBY (6), ZJ (7), GGI-221 within A. ferrooxidans (formerly Acidithiobacillus sp.) GGI-221 (8), Acidithiobacillus sp. SH (9), A. ferrooxidans strains ATCC 23270 (10), ATCC 53993 (11), Hel18 (12), YQH-1 (13), Acidithiobacillus sp. (formerly A. ferrooxidans) BY0502 (14), A. ferrivorans strains CF27 (15), PRJEB5721 (16), SS3 (17), YL15 (18), A. thiooxidans strains A01 (19), A02 (20), BY-02 (21), CLST (22), DMC (23), DXS-W (24), GD1-3 (25), JYC-17 (25), Licanantay (27), and ZBY (28). (DOC 37024 kb) [file 12864_2019_5827_MOESM1_ESM.doc]

**Supplemental Material for**

**Phylogeny, Divergent Evolution, and Speciation of Sulfur-Oxidizing *Acidithiobacillus* Populations**

Xian Zhang,^1*^ Xueduan Liu,^2,3^ Liangzhi Li,^2^ Guanyun Wei,^4^ Danli Zhang,^5^ Yili Liang,^2,3^ Bo Miao^2,3^

^*^Correspondence: Xian Zhang (zixuange2010@126.com)

^1^Department of Occupational and Environmental Health, Xiangya School of Public Health, Central South University, Changsha, China.

^2^School of Minerals Processing and Bioengineering, Central South University, Changsha, China.

^3^Key Laboratory of Biometallurgy of Ministry of Education, Central South University, Changsha, China.

^4^School of Life Sciences, Nantong University, Nantong, China.

^5^Department of Biology, Taiyuan Normal University, Taiyuan, China.

**Supplemental Figures**

Additional file 1: Figure S1 Geographic distributions and genome attributes of *Acidithiobacillus* strains used for the construction of genome-based phylogeny and the calculation of average nucleotide identity.


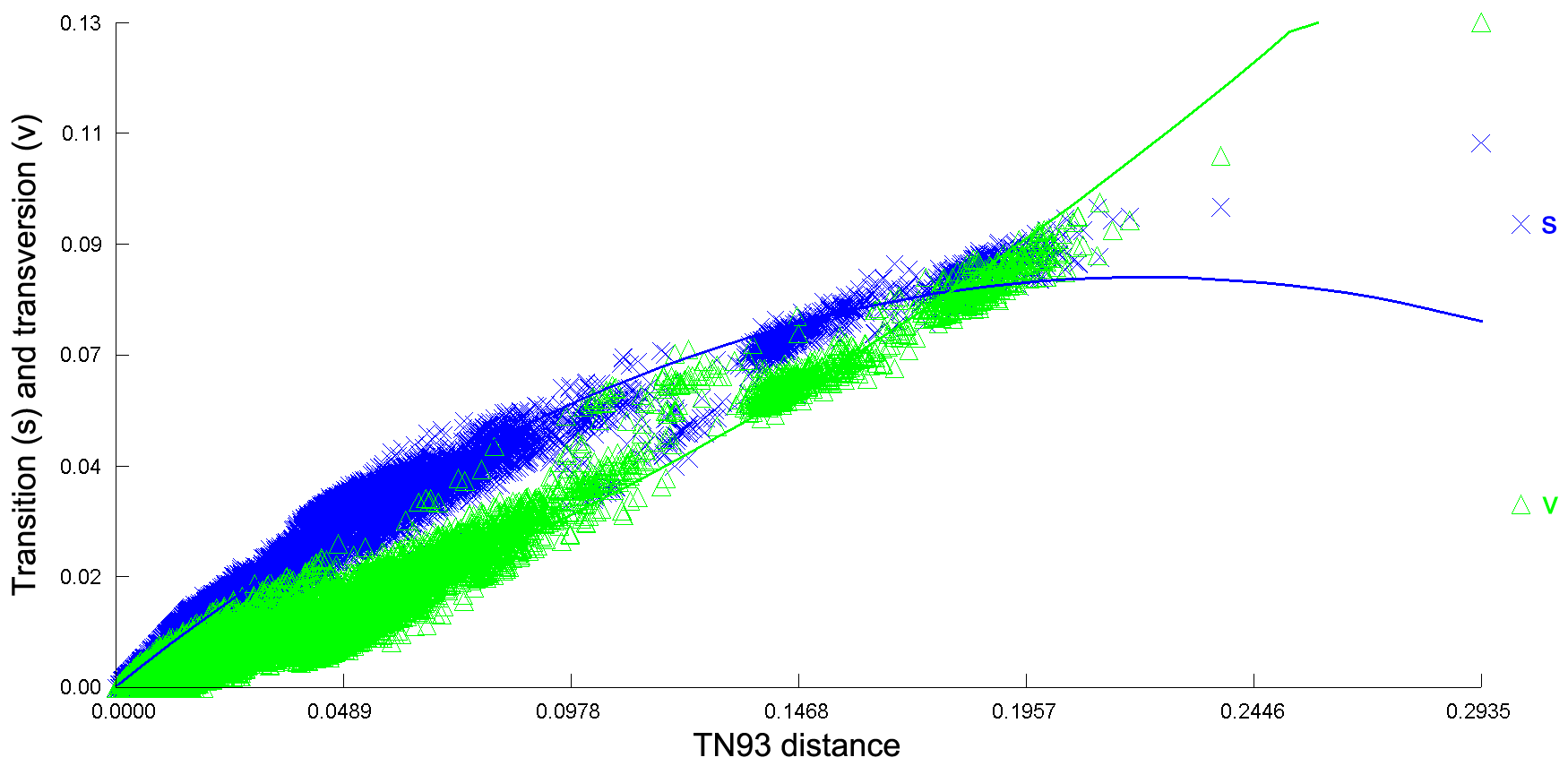


Additional file 1: Figure S2 Substitution pattern of 16S rRNA genes used for phylogenetic tree. The number of transitions (cross) and transversions (triangle) against the TN93 distance is shown in different colors. Each point in individual sites indicates a pairwise comparison between two of taxa.


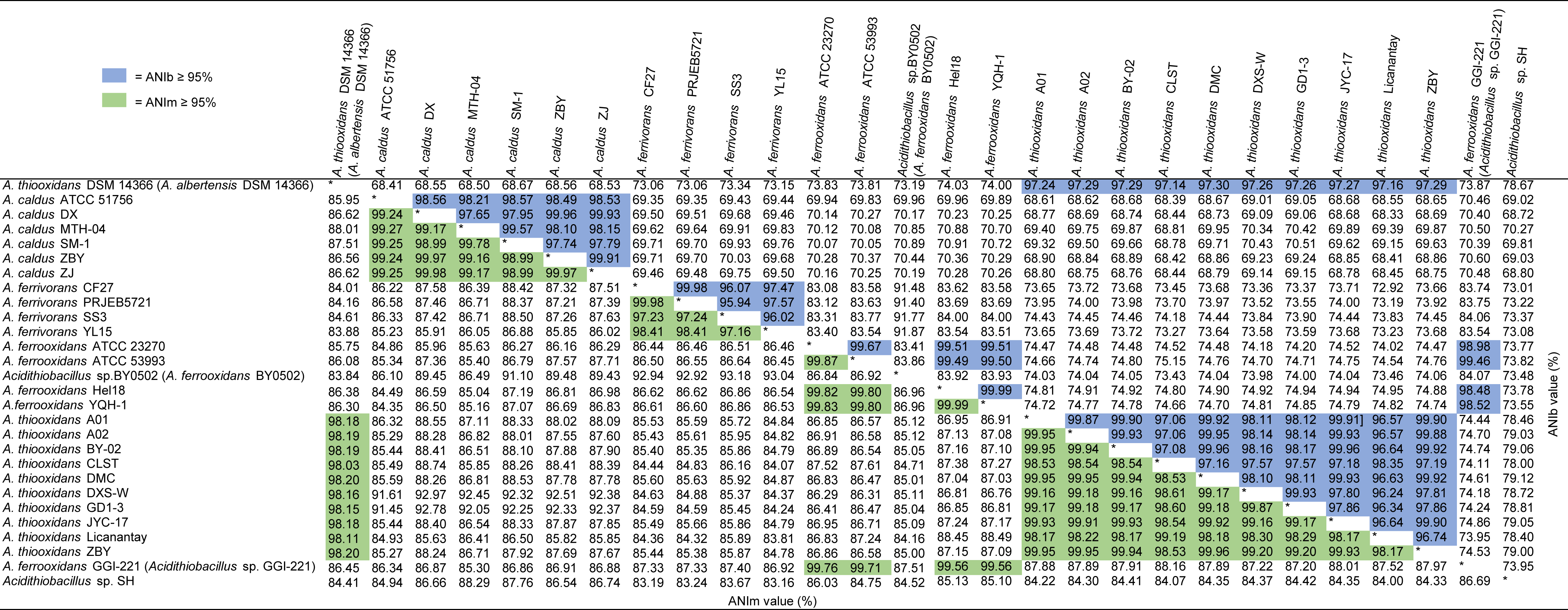


Additional file 1: Figure S3 Calculations of ANIb (upper half) and ANIm (lower half) for *Acidithiobacillus* strains. The values of ANI above the threshold for species delineation (95%) are highlighted.

**Supplementary Tables**

Additional file 1: Table S1. Statistics for the number of RNA (rRNA and tRNA) in *Acidithiobacillus* (*A.*) strains with available genomes.

| **Organism** | **rRNA** | | | **tRNA** |
| --- | --- | --- | --- | --- |
|  | **5S** | **16S** | **23S** |  |
| *A. caldus* | | | | |
| ATCC 51756^T*^ | 2 | 2 | 2 | 54 |
| ZBY | 1 | 1 | 1 | 53 |
| ZJ | 1 | 1 | 1 | 51 |
| DX | 1 | 1 | 1 | 51 |
| SM-1 | 2 | 2 | 2 | 55 |
| MTH-04 | 4 | 4 | 4 | 58 |
| *A. ferrooxidans* | | | | |
| ATCC 23270^T*^ | 2 | 2 | 2 | 88 |
| YQH-1 | 2 | 0 | 0 | 52 |
| Hel18 | 1 | 1 | 1 | 53 |
| ATCC 53993 | 2 | 2 | 2 | 50 |
| BY0502 | 1 | 1 | 1 | 52 |
| *A. ferrivorans* | | | | |
| SS3 | 2 | 2 | 2 | 51 |
| YL15 | 1 | 1 | 1 | 51 |
| PRJEB5721 | 2 | 1 | 2 | 84 |
| CF27 | 1 | 1 | 1 | 82 |
| *A. thiooxidans* | | | | |
| Licanantay | 2 | 0 | 1 | 94 |
| CLST | 1 | 1 | 1 | 79 |
| GD1-3 | 2 | 1 | 1 | 82 |
| DXS-W | 2 | 1 | 1 | 81 |
| A02 | 2 | 1 | 1 | 105 |
| BY-02 | 2 | 1 | 1 | 126 |
| ZBY | 2 | 1 | 1 | 126 |
| A01 | 1 | 1 | 1 | 127 |
| DMC | 2 | 1 | 1 | 126 |
| JYC-17 | 2 | 1 | 1 | 126 |
| *A. albertensis* | | | | |
| DSM 14366^T*^ | 2 | 2 | 2 | 50 |
| *Acidithiobacillus* sp. | | | | |
| GGI-221 | 1 | 1 | 1 | 83 |
| SH | 1 | 1 | 1 | 51 |

^*^ Type strain in any given species.

**Additional file 1: Table S3 Functional classifications of the common genes shared by *Acidithiobacillus* strains using an online platform KAAS.**

| **Pathway Number** | **Description** | **CDS Number** |
| --- | --- | --- |
| **Carbohydrate metabolism** | | **81** |
| ko00010 | Glycolysis / Gluconeogenesis | 11 |
| ko00020 | Citrate cycle (TCA cycle) | 7 |
| ko00030 | Pentose phosphate pathway | 10 |
| ko00040 | Pentose and glucuronate interconversions | 2 |
| ko00051 | Fructose and mannose metabolism | 5 |
| ko00052 | Galactose metabolism | 2 |
| ko00500 | Starch and sucrose metabolism | 5 |
| ko00520 | Amino sugar and nucleotide sugar metabolism | 11 |
| ko00620 | Pyruvate metabolism | 10 |
| ko00630 | Glyoxylate and dicarboxylate metabolism | 9 |
| ko00640 | Propanoate metabolism | 3 |
| ko00650 | Butanoate metabolism | 1 |
| ko00660 | C5-Branched dibasic acid metabolism | 3 |
| ko00562 | Inositol phosphate metabolism | 2 |
| **Energy metabolism** | | **55** |
| ko00190 | Oxidative phosphorylation | 21 |
| ko00195 | Photosynthesis | 7 |
| ko00710 | Carbon fixation in photosynthetic organisms | 11 |
| ko00720 | Carbon fixation pathways in prokaryotes | 6 |
| ko00680 | Methane metabolism | 7 |
| ko00910 | Nitrogen metabolism | 2 |
| ko00920 | Sulfur metabolism | 1 |
| **Lipid metabolism** | | **16** |
| ko00061 | Fatty acid biosynthesis | 5 |
| ko00071 | Fatty acid degradation | 1 |
| ko00561 | Glycerolipid metabolism | 3 |
| ko00564 | Glycerophospholipid metabolism | 6 |
| ko00600 | Sphingolipid metabolism | 1 |
| **Nucleotide metabolism** | | **46** |
| ko00230 | Purine metabolism | 27 |
| ko00240 | Pyrimidine metabolism | 19 |
| **Amino acid metabolism** | | **80** |
| ko00250 | Alanine, aspartate and glutamate metabolism | 6 |
| ko00260 | Glycine, serine and threonine metabolism | 10 |
| ko00270 | Cysteine and methionine metabolism | 17 |
| ko00280 | Valine, leucine and isoleucine degradation | 1 |
| ko00290 | Valine, leucine and isoleucine biosynthesis | 5 |
| ko00300 | Lysine biosynthesis | 10 |
| ko00220 | Arginine biosynthesis | 7 |
| ko00330 | Arginine and proline metabolism | 5 |
| ko00340 | Histidine metabolism | 3 |
| ko00350 | Tyrosine metabolism | 2 |
| ko00360 | Phenylalanine metabolism | 2 |
| ko00400 | Phenylalanine, tyrosine and tryptophan biosynthesis | 12 |
| **Metabolism of other amino acids** | | **13** |
| ko00410 | beta-Alanine metabolism | 1 |
| ko00450 | Selenocompound metabolism | 3 |
| ko00471 | D-Glutamine and D-glutamate metabolism | 1 |
| ko00473 | D-Alanine metabolism | 1 |
| ko00480 | Glutathione metabolism | 7 |
| **Glycan biosynthesis and metabolism** | | **23** |
| ko00531 | Glycosaminoglycan degradation | 1 |
| ko00540 | Lipopolysaccharide biosynthesis | 15 |
| ko00550 | Peptidoglycan biosynthesis | 7 |
| **Metabolism of cofactors and vitamins** | | **57** |
| ko00730 | Thiamine metabolism | 4 |
| ko00740 | Riboflavin metabolism | 3 |
| ko00750 | Vitamin B6 metabolism | 4 |
| ko00760 | Nicotinate and nicotinamide metabolism | 4 |
| ko00770 | Pantothenate and CoA biosynthesis | 6 |
| ko00780 | Biotin metabolism | 5 |
| ko00785 | Lipoic acid metabolism | 2 |
| ko00790 | Folate biosynthesis | 8 |
| ko00670 | One carbon pool by folate | 5 |
| ko00860 | Porphyrin and chlorophyll metabolism | 9 |
| ko00130 | Ubiquinone and other terpenoid-quinone biosynthesis | 7 |
| **Metabolism of terpenoids and polyketides** | | **11** |
| ko00900 | Terpenoid backbone biosynthesis | 6 |
| ko00523 | Polyketide sugar unit biosynthesis | 4 |
| ko01055 | Biosynthesis of vancomycin group antibiotics | 1 |
| **Biosynthesis of other secondary metabolites** | | **22** |
| ko00950 | Isoquinoline alkaloid biosynthesis | 1 |
| ko00960 | Tropane, piperidine and pyridine alkaloid biosynthesis | 2 |
| ko00332 | Carbapenem biosynthesis | 2 |
| ko00261 | Monobactam biosynthesis | 3 |
| ko00521 | Streptomycin biosynthesis | 6 |
| ko00525 | Acarbose and validamycin biosynthesis | 2 |
| ko00401 | Novobiocin biosynthesis | 2 |
| ko00405 | Phenazine biosynthesis | 2 |
| ko00333 | Prodigiosin biosyntheses | 2 |
| **Xenobiotics biodegradation and metabolism** | | **9** |
| ko00364 | Fluorobenzoate degradation | 1 |
| ko00361 | Chlorocyclohexane and chlorobenzene degradation | 1 |
| ko00623 | Toluene degradation | 1 |
| ko00980 | Metabolism of xenobiotics by cytochrome P450 | 1 |
| ko00982 | Drug metabolism - cytochrome P450 | 1 |
| ko00983 | Drug metabolism - other enzymes | 4 |

Additional file 1: Table S4 Summary for gene families in the genomes of *Acidithiobacillus* strains, including *A. thiooxidans* (formerly *A. albertensis*) DSM 14366 (1), *A. caldus* strains ATCC 51756 (2), DX (3), MTH-04 (4), SM-1 (5), ZBY (6), ZJ (7), GGI-221 within *A. ferrooxidans* (formerly *Acidithiobacillus* sp.) GGI-221 (8), *Acidithiobacillus* sp. SH (9), *A. ferrooxidans* strains ATCC 23270 (10), ATCC 53993 (11), Hel18 (12), YQH-1 (13), *Acidithiobacillus* sp. (formerly *A. ferrooxidans*) BY0502 (14), *A. ferrivorans* strains CF27 (15), PRJEB5721 (16), SS3 (17), YL15 (18), *A. thiooxidans* strains A01 (19), A02 (20), BY-02 (21), CLST (22), DMC (23), DXS-W (24), GD1-3 (25), JYC-17 (25), Licanantay (27), and ZBY (28).

| **No.** | **Orthogroups** | **Genes in orthogroups** | **Singletons/genes in genome** |
| --- | --- | --- | --- |
| 1 | 2888 | 3211 | 142 |
| 2 | 2421 | 2753 | 96 |
| 3 | 2626 | 2853 | 8 |
| 4 | 2414 | 2680 | 33 |
| 5 | 2655 | 3084 | 50 |
| 6 | 2640 | 2893 | 9 |
| 7 | 2642 | 2876 | 8 |
| 8 | 2607 | 3172 | 835 |
| 9 | 2489 | 2681 | 163 |
| 10 | 2627 | 2810 | 99 |
| 11 | 2479 | 2746 | 33 |
| 12 | 2408 | 2942 | 13 |
| 13 | 2706 | 2944 | 12 |
| 14 | 2709 | 2607 | 215 |
| 15 | 2972 | 3210 | 26 |
| 16 | 2996 | 3281 | 15 |
| 17 | 2568 | 2810 | 206 |
| 18 | 2480 | 2661 | 117 |
| 19 | 3333 | 3705 | 15 |
| 20 | 3258 | 3633 | 7 |
| 21 | 3308 | 3676 | 8 |
| 22 | 3146 | 3523 | 97 |
| 23 | 3364 | 3765 | 8 |
| 24 | 3316 | 3823 | 11 |
| 25 | 3317 | 3813 | 12 |
| 26 | 3354 | 3732 | 6 |
| 27 | 3253 | 3662 | 112 |
| 28 | 3333 | 3707 | 8 |

**Supplementary References**

Liljeqvist M, Valdes J, Holmes DS, Dopson M. (2011). Draft genome of the psychrotolerant acidophile *Acidithiobacillus ferrivorans* SS3. *J Bacteriol* **193:** 4304-4305.

Liu X, Lin J, Zhang Z, Bian J, Zhao Q, Liu Y *et al*. (2007). Construction of conjugative gene transfer system between *E. coli* and moderately thermophilic, extremely acidophilic *Acidithiobacillus caldus* MTH-04. *J Microbiol Biotechnol* **17:** 162-167.

Peng T, Ma L, Feng X, Tao J, Nan M, Liu Y *et al*. (2017). Genomic and transcriptomic analyses reveal adaptation mechanisms of an *Acidithiobacillus ferrivorans* strain YL15 to alpine acid mine drainage. *PLoS One* **12:** e0178008.

Talla E, Hedrich S, Mangenot S, Ji B, Johnson DB, Barbe V *et al*. (2014). Insights into the pathways of iron-and sulfur-oxidation, and biofilm formation from the chemolithotrophic acidophile *Acidithiobacillus ferrivorans* CF27. *Res Microbiol* **165:** 753-760.

Travisany D, Cortés MP, Latorre M, Di Genova A, Budinich M, Bobadilla-Fazzini RA *et al*. (2014). A new genome of *Acidithiobacillus thiooxidans* provides insights into adaptation to a bioleaching environment. *Res Microbiol* **165:** 743-752.

Valdés J, Pedroso I, Quatrini R, Dodson RJ, Tettelin H, Blake R *et al*. (2008). *Acidithiobacillus ferrooxidans* metabolism: from genome sequence to industrial applications. *BMC Genomics* **9:** 597.

Valdes J, Quatrini R, Hallberg K, Dopson M, Valenzuela PDT, Holmes DS. (2009). Draft genome sequence of the extremely acidophilic bacterium *Acidithiobacillus caldus* ATCC 51756 reveals metabolic versatility in the genus *Acidithiobacillus*. *J Bacteriol* **191:** 5877-5878.

Yan L, Zhang S, Wang W, Hu H, Wang Y, Yu G *et al*. (2015). Draft genome sequence of *Acidithiobacillus ferrooxidans* YQH-1. *Genomics Data* **6:** 269-270.

Yin H, Zhang X, Liang Y, Xiao Y, Niu J, Liu X. (2014). Draft genome sequence of the extremophile *Acidithiobacillus thiooxidans* A01, isolated from the wastewater of a coal dump. *Genome Announc* **2:** e00222-14.

You XY, Guo X, Zheng HJ, Zhang MJ, Liu LJ, Zhu YQ *et al*. (2011). Unraveling the *Acidithiobacillus caldus* complete genome and its central metabolisms for carbon assimilation. *J Genet Genomics* **38:** 243-252.

Zhang X, Feng X, Tao J, Ma L, Xiao Y, Liang Y *et al*. (2016a). Comparative genomics of the extreme acidophile *Acidithiobacillus* *thiooxidans* reveals intraspecific divergence and niche adaptation. *Int J Mol Sci* **17:** 1355.

Zhang X, Liu X, He Q, Dong W, Zhang X, Fan F *et al*. (2016b). Gene turnover contributes to the evolutionary adaptation of *Acidithiobacillus* *caldus*: insights from comparative genomics. *Front Microbiol* **7:** 1960.

Zhang X, Liu Z, Wei G, Yang F, Liu X. (2018). *In silico* genome-wide analysis reveals the potential links between core genome of *Acidithiobacillus thiooxidans* and its autotrophic lifestyle. *Front Microbiol* **9:** 1255.
